# Patient Mobilisation Attitudes and Beliefs Survey‐Intensive Care Unit: Turkish Validation and Reliability Study

**DOI:** 10.1002/nop2.70512

**Published:** 2026-03-31

**Authors:** Sevgi Uzuntepe, Imatullah Akyar

**Affiliations:** ^1^ Ankara Training and Research Hospital Ankara Turkey; ^2^ Graduate School of Health Sciences Hacettepe University Ankara Turkey; ^3^ Faculty of Nursing, Hacettepe University Ankara Turkey

**Keywords:** barrier, critical care, health personnel, mobilisation, validity and reliability

## Abstract

**Purpose:**

To assess the psychometric properties of the Turkish ‘Patient Mobilization Attitudes & Beliefs Survey‐ICU,’ which was developed to identify the perceived barriers of healthcare professionals in mobilising patients in intensive care units.

**Methods:**

The study was conducted from 3 December 2021 to 21 October 2023, methodologically. A total of 180 healthcare professionals, including 163 nurses, 12 physicians and five physiotherapists working in adult intensive care units of three tertiary hospitals in Türkiye, were recruited for the study. This study used translation‐back‐translation, the Davis method, confirmatory factor analysis, Cronbach's alpha and split‐half test for validity and reliability of the scale.

**Results:**

The validity and reliability analyses revealed the removal of the 5th, 6th, 13th and 20th items from the original scale, and a 22‐item scale structure with two subscales was obtained. The Spearman‐Brown coefficient was 0.799, and the Guttman coefficient was calculated as 0.782. The Cronbach alpha coefficient of the scale is 0.818; of the knowledge and attitude subscales is 0.721, and of the behaviour subscale is 0.708.

**Conclusion:**

The psychometric characteristics of the Turkish Patient Mobilization Attitudes & Beliefs Survey‐ICU are a valid and reliable tool for determining the barriers healthcare professionals perceive about the mobilisation of intensive care patients.

**Implications for the Profession and/or Patient Care:**

Assessment of barriers to mobilisation of ICU patients will help nurses mobilise patients safely in the care routine, which will help reduce immobilisation complications.

**Impact:**

The use of scales in the evaluation of mobilisation in intensive care is limited. As a result of the research, the ‘Patient Mobilization Attitudes & Beliefs Survey‐ICU’ scale was found to be a valid and reliable tool. This scale is important for healthcare professionals and patients to increase the mobilisation of intensive care patients.

**Reporting Method:**

The study was reported according to the EQUATOR GRRAS guideline.

**Patient or Public Contribution:**

The study recruited healthcare professionals, including 163 nurses, 12 physicians and 5 physiotherapists, who were actively working in adult intensive care units. The study data was collected with ‘Participant Information Form’ and ‘Patient Mobilization Attitudes & Beliefs Survey‐ICU’. Participants were invited to engage in face‐to‐face interviews. Following an explanation of the study's objectives and criteria, verbal and written consent were obtained from 180 eligible healthcare professionals who volunteered to participate and completed the data collection forms.

## 1 Introduction

ICU patients are often immobile in bed due to the course of their illness, medications (e.g., sedation) and devices (e.g., continuous renal replacement therapy) (Castro‐Avila et al. 2015). Efforts to integrate early mobilisation into daily routine care practices have been extensive (Miranda Rocha et al. 2017), as studies demonstrate its numerous positive effects (Alaparthi et al. 2020; Nydahl et al. 2022; Zang et al. 2020; Zhang et al. 2019; Wang et al. 2020). Early mobilisation is a patient‐centred approach that focuses on the patient's functionality, which has benefits such as preserving and restoring the patient's musculoskeletal strength and function (Hodgson et al. 2013), reducing the duration of mechanical ventilation and the length of hospital stay with its positive effects (Alaparthi et al. 2020). A primary goal of ICU care is to preserve patient mobility and functional capacity, typically by aiming for mobilisation at least twice daily once hemodynamic stability is achieved (AHRQ 2021). The optimal timing for initiating mobilisation varies depending on the patient's general condition, ranging from within the first 24 h of mechanical ventilation to within the first week of ICU admission (Ding et al. 2019; Renner et al. 2023).

Despite the recognised importance, early mobilisation practices remain inconsistently implemented within ICUs, often leading to delays in patient mobilisation for various reasons (Goodson et al. 2020). Multiple barriers impede the widespread adoption of mobilisation protocols at various levels, encompassing patient‐related factors, healthcare professionals' perceptions and attitudes, as well as institutional and unit cultures (Lewis et al. 2023). Identified barriers to early mobilisation may also include inadequate administrative support for mobilisation programming (Hsu et al. 2020), insufficiencies in personnel and equipment (Potter et al. 2021) and deficiencies in the coordination of interprofessional perspectives and procedural application (Dubb et al. 2016). Studies within the literature consistently highlight the perceived barriers among healthcare professionals—pertaining to knowledge, attitude and behaviour—that may impede the mobilisation of intensive care patients (Akhtar and Deshmukh 2021; Goodson et al. 2020; Lewis et al. 2023).

Health professionals' perceptions of barriers across various dimensions significantly influence early mobilisation practices (Alaparthi et al. 2020). According to knowledge, attitude and practice theory, attitudes gradually form after systematically examining acquired knowledge, transforming knowledge into beliefs that guide human behaviour (Schrader and Lawless 2004). Sustainable changes in behaviour are therefore more likely to occur when knowledge and attitudes are first developed, rather than by targeting behaviour alone. However, even when knowledge and attitude barriers are adequately addressed, behavioural barriers may still hinder implementation. To achieve successful change, it is necessary to assess barriers using validated measures and then design educational strategies to address the identified gaps (Cabana et al. 1999).

In intensive care units, specific barriers to mobilisation have been documented. These barriers in intensive care units encompass the competence and knowledge of healthcare professionals regarding the risks and benefits of early mobilisation (Hsu et al. 2020), negative attitudes among both families and healthcare professionals towards patient mobilisation, inadequate utilisation of objective assessment tools (Potter et al. 2021), deficiencies or delays in assessing patients' suitability for mobilisation, as well as challenges in coordinating interprofessional opinions and procedural application (Dubb et al. 2016), and confusion regarding interdisciplinary roles or absence of a cohesive multidisciplinary team (Potter et al. 2021). Addressing these barriers to mobilisation has been demonstrated to be effectively achieved through clear communication and recognition of the importance of early mobilisation, coupled with concerted multidisciplinary efforts (Hashem et al. 2016).

Numerous studies conducted worldwide have identified barriers to patient mobilisation in intensive care settings (Dubb et al. 2016; Harrold et al. 2015; Lim et al. 2023; Mudge et al. 2020). However, recent investigations on the mobilisation of intensive care patients within our country predominantly centre on the assessment of patients' mobilisation status (Ibrahimoğlu et al. 2022), the impact of early mobilisation on delirium (Karadaş and Özdemir 2019; Kılıç and Kav 2022), and stroke (Salcı et al. 2019), as well as the development of mobilisation protocols for post‐operative patients (Aygin et al. 2022; Meşe et al. 2023).

In the Turkish ICU context, additional barriers may relate to staffing levels and cultural attitudes towards family involvement. Nursing availability and time are common institutional barriers to early mobilisation (Najjar et al. 2021). A national study conducted reported that more than half of the nurses (69.4%) care for an average of 1–4 patients per day (Özel et al. 2011), a workload that is somewhat higher than the legislation limits (Ministry of Health 2011). Family participation also tends to be limited; in Turkish ICUs, 80.8% of family members do not take part in patient care, often due to institutional policies (51.4%) and families' concerns about causing harm to patients (48.6%) (Yeşilyurt et al. 2024). Since mobilisation practices are influenced by factors such as nurse staffing levels (Bakhru et al. 2016), equipment availability (Barber et al. 2015) and cultural norms, the need to adapt the scale is essential. Higher patient‐to‐nurse ratios, limited resources (appropriate equipment and staff), and restricted family involvement in patient care may all contribute to shaping healthcare professionals' perceptions of mobilisation. To our knowledge, there is a notable scarcity of research focusing on the experiences and perspectives of ICU professionals regarding patient mobilisation, as well as a dearth of tools dedicated to identifying barriers to patient mobilisation.

## 2 The Study

### 2.1 Aim and Objective

The aim of this study was to assess the Turkish validity and reliability of the ‘Patient Mobilization Attitudes & Beliefs Survey‐ICU’. This instrument was developed by Hoyer et al. to identify the perceived barriers among healthcare professionals concerning patient mobilisation in intensive care units.

## 3 Methods

### 3.1 Design

To assess the psychometric properties of the Turkish ‘Patient Mobilization Attitudes & Beliefs Survey‐ICU’, a methodological study with cross‐sectional design was used.

### 3.2 Sampling and Recruitment

The study was conducted within the adult intensive care units of three tertiary hospitals, from 3 December 2021, to 21 October 2023. Two of the participating institutions were university hospitals, while the third was a training and research hospital. These facilities collectively comprised 28 adult intensive care units across the first, second and third levels, with a total of 251 beds. Notably, none of the institutions had implemented a formal mobility protocol at the time of the study. The study recruited healthcare professionals, including nurses, physicians and physiotherapists, who were actively working in adult intensive care units.

### 3.3 Sample Size

Guidelines recommend sampling 5–10 times the number of items in a scale for validity and reliability studies (Alpar 2014). Given that the ‘Patient Mobilization Attitudes & Beliefs Survey‐ICU’ comprised 26 items, a minimum sample size of 130 participants was deemed necessary‐equivalent to 5 times the number of scale items.

### 3.4 Population and Sample

The research population encompassed 295 nurses, 39 physicians and five physiotherapists employed within the adult intensive care units of the three participating institutions. Ultimately, the sample for the study comprised 180 healthcare professionals (163 nurses, 12 physicians and 5 physiotherapists) who met the inclusion criteria and consented to participate between 24 January 2023 and 20 October 2023.

### 3.5 Inclusion or Exclusion Criteria

Inclusion criteria for nurses were holding a bachelor's degree and possessing a minimum of 3 months' experience in ICU, while physicians and physiotherapists were required to have at least 1 month of relevant experience in adult intensive care units. These cutoff periods for experience were determined in accordance with the clinical orientation policies and processes of the respective institutions. Additionally, 3‐month threshold is also methodologically supported by nursing transition theories; transition from the ‘Novice’ to ‘Advanced Beginner’ stage, ensuring that participants have moved past the transition shock and initial clinical socialisation to provide a stable and reliable assessment of workplace barriers (Kramer 1974; Benner 1984; Duchscher and Windey 2018).

### 3.6 Data Collection

Data were collected using the ‘Participant Information Form’ and the ‘Patient Mobilization Attitudes & Beliefs Survey‐ICU’. The ‘Participant Information Form’ queried socio‐demographic data and data on clinical experience on patient mobilisation.

The ‘Patient Mobilization Attitudes & Beliefs Survey‐ICU’ scale was originally developed by Goodson et al. in 2018 at Johns Hopkins University, drawing upon the study conducted by Hoyer et al. (2015) in the general clinics of Johns Hopkins University. The scale, initially crafted in English, is administered through self‐report. Comprising 26 items, the scale is structured into three subscales: ‘knowledge’ (four items), ‘attitude’ (nine items) and ‘behaviour’ (13 items). The knowledge subscale evaluates healthcare professionals' training and education regarding patient mobilisation, while the attitude subscale delineates professionals' self‐efficacy levels towards mobilisation efforts and their perceptions of colleagues' attitudes. The behaviour subscale addresses practical constraints and external barriers that impede the implementation of patient mobilisation by healthcare professionals. Following the 26 items, the scale concludes with an open‐ended question, providing respondents with an opportunity to raise additional issues pertaining to patient mobilisation that may not have been addressed within the structured items. Each of the 26 items is scored on a 5‐point Likert scale, ranging from 1 (strongly disagree) to 5 (strongly agree). Notably, items 1, 3, 4, 7, 10, 12, 13, 15, 17, 19, 21 and 23 are reverse‐coded. The scores obtained from the scale are then transformed into a 0–100 scoring system. Higher total and subscale scores indicate a greater perceived level of barriers to patient mobilisation.

Participants were invited to engage in face‐to‐face interviews. After explaining the study's objectives and criteria, verbal and written consent were obtained from 180 eligible healthcare professionals who volunteered to participate. Subsequently, participants were requested to complete the data collection forms. On average, each participant spent approximately 7–8 min completing the data collection tools.

### 3.7 Data Analysis

The descriptive data were analysed using descriptive statistics, including measures such as mean, standard deviation, percentage, total and minimum and maximum values. No missing data were observed across the survey items.

The validation and reliability assessment of the scale entailed the following steps:

- The translation‐back‐translation method was employed to assess the language validity of the scale (Karaçam 2019).
- Expert opinions were consulted to evaluate the content validity of the scale, utilising the Davis method for assessment.
- Confirmatory factor analysis was conducted to evaluate the construct validity of the scale.
- Reliability was assessed using the split‐half testing method.

#### 3.7.1 Language Validity

The ‘Patient Mobilization Attitudes & Beliefs Survey‐ICU’ scale was translated from English into Turkish by three nursing faculty members (specialising in internal medicine nursing and nursing principles), all native Turkish speakers. Each expert independently translated the scale, and the researchers subsequently compared and synthesised their translations into a unified version with necessary adjustments. The phrase ‘My patients are too sick to be mobilized’ was adapted to ‘My patients are too severe to be mobilized’. Because the translations were largely consistent, only slight modifications were required. The draft Turkish version was then reviewed by a Turkish language expert to ensure clarity and conformity with spelling rules. Following this, the scale was back‐translated into English by two bilingual nursing faculty members (specialising in internal medicine and surgical nursing). The back‐translated version was compared with the original scale, and necessary changes were made. The finalised version was submitted to one of the developers of the original scale for review. Finally, a pilot study with 10 nurses was conducted to evaluate the linguistic clarity before proceeding to the content validity phase.

#### 3.7.2 Content Validity

Following the establishment of language validity, the content validity of the scale was assessed using the Davis method with 11 experts: three nursing faculty, three intensivists, three physiotherapists, an intensive care nurse, and an occupational therapist faculty member. Each expert rated the items on a 4‐point scale (‘1: not appropriate, 2: somewhat appropriate, 3: appropriate, 4: very appropriate’). For each item, the content validity ratio was calculated as the proportion of experts rating the item as ‘3’ or ‘4’, and the content validity index (CVI) was obtained by averaging content validity ratios across all items. The CVI should ideally be 0.80 or higher to ensure satisfactory content validity. In this study, the CVI was 0.87, indicating satisfactory content validity. Based on expert feedback, minor revisions were made to finalise the scale.

#### 3.7.3 Construct Validity

The scale's construct validity was evaluated using confirmatory factor analysis with the maximum likelihood technique. Model fit was assessed based on standard fit indices (*χ* ^2^/SD GFI, AGFI, RMSEA, SRMR, CFI, IFI, TLI) and individual item regression coefficients (estimates).

#### 3.7.4 Reliability Analysis

Internal consistency reliability was assessed using Cronbach's alpha. Values were interpreted as follows: ‘0.80–1.00: highly reliable’, ‘0.60–0.79: fairly reliable’, ‘0.40–0.59: low reliability’ and ‘0.00–0.39: unreliable’ (Alpar 2020). A coefficient ≥ 0.70 was considered acceptable for the total scale and subscales. Item‐total correlation coefficients were also examined, with values above +0.25 indicating sufficient reliability. In addition, the split‐half reliability was tested using Spearman‐Brown and Guttman formulas to compare consistency across coefficients. The participant characteristics and the group differences in total and subscale scores were examined with an independent sample *t*‐test and one‐way analysis of variance (ANOVA). Data was analysed using IBM SPSS (Statistical Package for the Social Sciences) version 23.0 and AMOS 23.0 (Analysis of Moment Structures) programmes.

### 3.8 Ethical Considerations

The study adhered to the principles outlined in the Helsinki Declaration. Permission to utilise the ‘Patient Mobilization Attitudes & Beliefs Survey‐ICU’ scale was obtained from the original developer. Ethical approval was granted by the Institutional Review Board Committee of the researchers' respective universities (Approval #: GO 21/1106, issued on 7 December 2021). Prior to participation, healthcare professionals provided written informed consent. Participants were not offered any financial or material incentives for their involvement in the study.

## 4 Results

### 4.1 Subjects

The mean age of the participants was 29.5 ± 5.43 years, with the majority (70.6%) within the age range of 21–30 years. A significant proportion of participants (81.7%) were female, and the vast majority (90.5%) were nurses, with 83.9% holding a bachelor's degree. On average, participants reported professional experience of 76.7 months (6.4 years), with specific experience in intensive care averaging 61.7 months (5.1 years), and tenure within their current unit averaging 48.7 months (4 years). Many participants (68.3%) were employed in third‐level ICUs, while half (55.6%) reported not having received training on patient mobilisation in the ICU. Additionally, half of the participants (50%) indicated the availability of a patient mobilisation protocol within their institution (Table 1).

**TABLE 1:** Participants' descriptive characteristics (*n* = 180).

	*n*	%
Age $\bar{x}$ ± SD (min–max) = 29.5 ± 5.43 (22–50)		
21–30	127	70.6
31–40	42	23.3
41–50	11	6.1
Sex		
Female	147	81.7
Male	33	18.3
Clinical role		
Nurse	163	90.5
Physician	12	6.7
Physiotherapist	5	2.8
Educational background		
Bachelor	151	83.9
Postgraduate (Master's and PhD)	29	16.1
Intensive care level		
Level 1	18	10.0
Level 2	39	21.7
Level 3	123	68.3
Patient mobilisation training		
Yes	80	44.4
No	100	55.6
Patient mobilisation protocol availability in participants' institution		
Yes	90	50.0
No	90	50.0

	$\bar{x}$ ± SD	Median (min–max)
Clinical experience in the profession (months)	76.7 ± 68.9	48 (3–288)
Clinical experience in current unit (months)	48.7 ± 49.1	36 (1–276)
Clinical experience in intensive care (months)	61.7 ± 59.9	36 (1–288)

*Note:* *n*: number, %: percentage, $\bar{x}$: mean, SD: standard error, min–max: minimum–maximum.

### 4.2 Validity

#### 4.2.1 Language Validity

Change was made to the scale item following the translation process (forward and back translation) and the assessment by Turkish language experts. Following the completion of the Turkish translation, a pilot study involving 10 nurses was conducted to assess the scale's linguistic comprehensibility. The pilot study confirmed the linguistic validity of the translated scale.

#### 4.2.2 Content Validity

During the content validity assessment, according to experts' feedback, adjustments were made to the introductory information section of the scale, specifically by removing the professional groups ‘nurse practitioner, clinical technician, respiratory therapist’ and refining the categorisation of doctors to ‘specialist, assistant, intern doctor’. The content validity index of the scale was 0.87.

#### 4.2.3 Construct Validity

**FIGURE 1:**
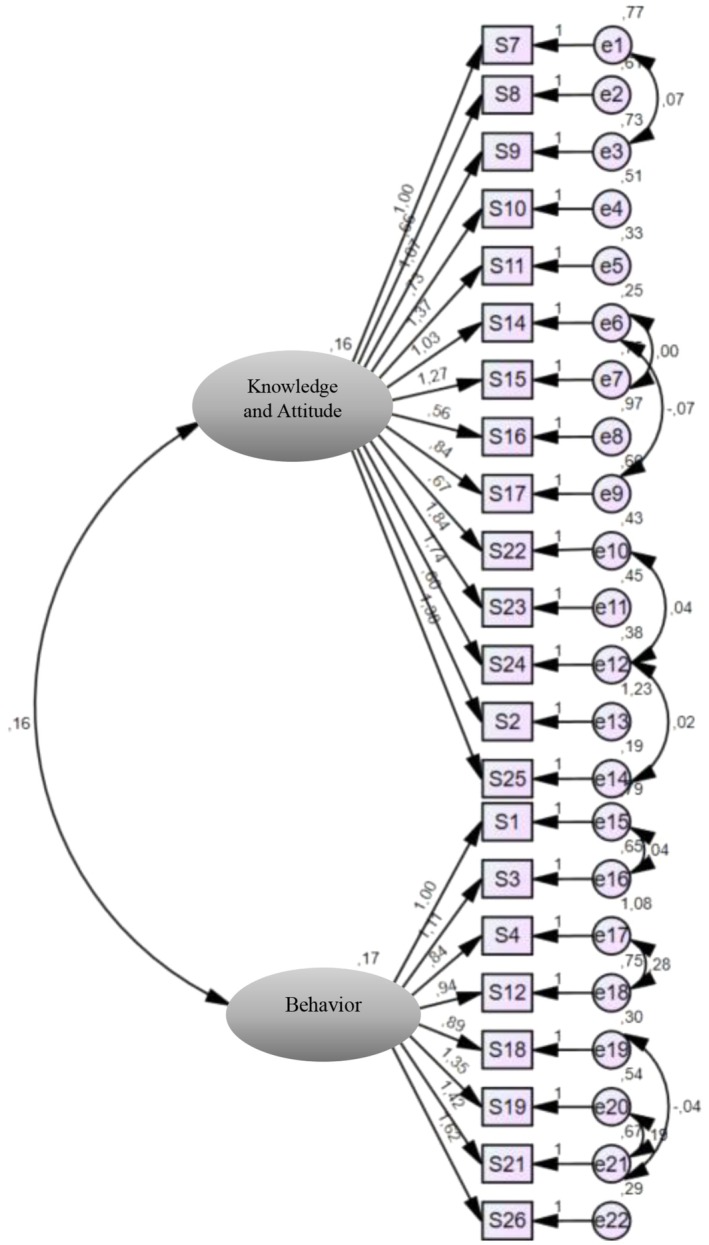
‘Patient Mobilization Attitudes & Beliefs Survey‐ICU’ scale structural equation model for confirmatory factor analysis (22 items, 2 subscales).

Confirmatory factor analysis was employed to assess the construct validity of the scale, focusing on fit indices and regression coefficients (estimates) of the items. The confirmatory factor analysis indicated *χ* ^2^/SD of 2.555, falling within the acceptable fit range. However, other fit indices, including GFI (0.730), AGFI (0.679), RMSEA (0.093), SRMR (0.084), CFI (0.577), IFI (0.589) and TLI (0.534), did not meet recommended thresholds for ‘acceptable/good fit’. To improve model fit, modification indices were examined, and error covariances between theoretically related items were allowed where appropriate. Despite adjustments, the majority of fit indices remained outside the acceptable range. Item deletion led to improved fit indices in the subsequent analysis. Regression coefficients (estimates) for all items ranged from −0.473 to 7.406. Items 5, 6, 13 and 20 exhibited non‐significant regression coefficients (*p* > 0.05) and were consequently removed from the scale. Subsequent confirmatory factor analysis with the revised scale comprising 22 items revealed fit index values within the ‘acceptable/good fit value’ range: *χ* ^2^/SD (1.286—good fit), GFI (0.915—acceptable fit), AGFI (0.856—acceptable fit), RMSEA (0.040—good fit), SRMR (0.042—good fit), CFI (0.955—acceptable fit), IFI (0.958—good fit) and TLI (0.930—acceptable fit). Furthermore, all items demonstrated statistically significant regression coefficients (*p* < 0.05). Consequently, the analysis proceeded with the revised version of the scale consisting of 22 items and two subscales (Table 2, Figure 1).

**TABLE 2:** Confirmatory factor analysis (22 items, 2 subscales).

	Regression coefficient (estimate)	Standard error	Critical ratio	*p*
Knowledge – attitude subscale				
Item 2	0.602	0.252	2.393	0.017
Item 5	1.000	—	—	—
Item 6	0.659	0.235	2.807	0.005
Item 7	1.073	0.305	3.520	***
Item 8	0.734	0.228	3.218	0.001
Item 9	1.367	0.349	3.921	***
Item 11	1.031	0.264	3.911	***
Item 12	1.275	0.334	3.818	***
Item 13	0.562	0.257	2.186	0.029
Item 14	0.836	0.243	3.439	***
Item 18	0.675	0.228	2.959	0.003
Item 19	1.839	0.412	4.460	***
Item 20	1.743	0.447	3.901	***
Item 21	1.381	0.352	3.927	***
Behaviour subscale				
Item 1	1.000	—	—	—
Item 3	1.112	0.287	3.875	***
Item 4	0.835	0.272	3.074	0.002
Item 10	0.941	0.281	3.352	***
Item 15	0.894	0.246	3.639	***
Item 16	1.347	0.312	4.315	***
Item 17	1.416	0.364	3.891	***
Item 22	1.615	0.381	4.236	***

*** *p* < 0.001.

### 4.3 Reliability

The scale's internal consistency was assessed using the Cronbach's alpha coefficient. The Cronbach alpha coefficient for the entire scale (26 items, 3 subscales) was calculated as 0.796, indicating a satisfactory level of reliability. However, upon further examination of the subscales, the Cronbach's alpha coefficient for the knowledge subscale was 0.389, falling below the threshold for reliability. In contrast, the attitude subscale yielded a coefficient of 0.672 (fairly reliable), and the behaviour subscale yielded a coefficient of 0.682 (fairly reliable). The analysis indicated that the knowledge subscale lacked reliability, and the Cronbach's alpha coefficients for the attitude and behaviour subscales were below the acceptable threshold of 0.70. Upon evaluation of item‐total correlation coefficients, it was observed that items 5, 6, 13 and 20 exhibited coefficients below +0.25. Consequently, these items were removed from the scale to improve reliability. Subsequent internal consistency analysis of the revised scale, consisting of 22 items, revealed improved item‐total correlation coefficients exceeding +0.25. The Cronbach's alpha coefficients for the total scale and both subscales were 0.818 (high reliability) for the total scale, 0.721 (fairly reliable) for the knowledge and attitude subscales and 0.708 (fairly reliable) for the behaviour subscale. The results indicate satisfactory levels of internal consistency reliability for the total scale and its subscales. Additionally, split‐half testing yielded a Spearman‐Brown coefficient of 0.799 and a Guttman coefficient of 0.782, further affirming the internal consistency and reliability of the scale.

### 4.4 Scale Descriptive Results

The mean total score of the scale was 50.23 ± 9.82, with the knowledge and attitude subscale averaging at 50.83 ± 9.89, and the behaviour subscale at 49.19 ± 12.40. The item analysis revealed that nurses perceived the following barriers to patient mobilisation in intensive care as most prominent: ‘My patients often have contraindications to be mobilized’ (Item 8), ‘My patients are too sick to be mobilized’ (Item 1), ‘A physical therapist should be the primary care provider to mobilise my patients’ (Item 4), and ‘Increasing mobilization of my patients will be more work for nurses’(Item 10) (Table 3).

**TABLE 3:** ‘Patient Mobilisation Attitudes & Beliefs Survey‐ICU’ scale item analysis (*n* = 180) (22 items, 2 subscales).

Knowledge and attitude subscale	$\bar{x}$ ± SD	Response				
$\bar{x}$ ± SD (min–max) = 50.83 ± 9.89 (25.71–72.86)		Distributions (%)^b^				
		1	2	3	4	5
2. I have received training on how to safely mobilise my patients	3.36 ± 1.28	21.1	34.0	14.4	21.1	9.4
5. We don't have the proper equipment and/or furnishings to mobilise my patients^a^	3.26 ± 1.20	12.2	41.7	17.2	18.3	10.6
6. The physical functioning of my patients is regularly discussed between the patient's healthcare providers (nurses, physicians, physical therapists)	3.74 ± 0.99	21.1	48.9	15.6	12.2	2.2
7. Nurse‐to‐patient staffing is adequate to mobilise patients on my unit	2.94 ± 1.28	10.5	31.7	16.1	25.0	16.7
8. My patients often have contraindications to be mobilised^a^	2.59 ± 0.99	3.9	15.5	26.7	43.9	10.0
9. Unless there is a contraindication, my patients are mobilised at least once daily by Nurses	4.11 ± 0.92	37.2	46.1	9.5	5.0	2.2
11. My leadership is very supportive of patient mobilisation	4.07 ± 0.74	27.2	56.7	12.8	2.8	0.5
12. Increasing the frequency of mobilising my patients increases my risk for injury^a^	3.13 ± 1.18	11.7	32.2	24.4	21.7	10.0
13. Patients who can be mobilised usually have appropriate physician orders to do so	3.43 ± 1.18	15.6	46.7	12.2	17.2	8.3
14. My patients are resistant to being mobilised^a^	3.07 ± 1.06	6.7	32.2	30.6	22.8	7.7
18. I document the physical functioning status of my patients during my shift/workday	4.23 ± 0.88	43.3	45.0	5.0	5.0	1.7
19. I do not have time to mobilise my patients during my shift/workday^a^	3.46 ± 1.10	19.4	31.7	28.3	16.7	3.9
20. Unless there is a contraindication, I mobilise my patients at least once during my shift/workday	3.99 ± 1.03	37.2	38.3	13.9	7.8	2.8
21. Unless there is a contraindication, I educate my patients to exercise or increase their physical activity while on my hospital unit	4.15 ± 0.81	36.1	47.8	11.7	3.9	0.5

Behaviour subscale	$\bar{x}$ ± SD	1	2	3	4	5
$\bar{x}$ ± SD (min–max) = 49.19 ± 12.40 (20–80)						
1. My patients are too sick to be mobilised^a^	2.86 ± 1.22	8.4	28.3	21.1	26.1	16.1
3. Increasing mobilisation of my patients will be harmful to them (e.g., falls, IV line removal)^a^	3.68 ± 1.11	24.4	41.1	17.8	11.7	5.0
4. A physical therapist should be the primary care provider to mobilise my patients^a^	2.56 ± 1.26	6.7	21.7	18.3	28.3	25.0
10. Increasing mobilisation of my patients will be more work for nurses^a^	2.54 ± 1.22	5.0	21.1	22.8	25.5	25.5
15. I believe that my patients who are mobilised at least once daily (if there is no contraindication) will have better outcomes	4.33 ± 0.73	46.1	43.9	7.2	2.8	—
16. I am not sure when it is safe to mobilise my patients^a^	3.66 ± 1.08	22.2	41.7	21.1	10.0	5.0
17. I do not feel confident in my ability to mobilise my patients^a^	3.98 ± 1.17	41.1	37.8	6.1	8.9	6.1
22. My patients have time during their day to be mobilised at least once daily	4.03 ± 0.93	35.6	40.5	16.7	6.1	1.1

*Note:* %: percentage, $\bar{x}$: mean, SD: standard error, min–max: minimum–maximum.

^a^ The 1st, 3rd, 4th, 5th, 8th, 10th, 12th, 14th, 16th, 17th, 19th items of the scale are reverse coded.

^b^ Items are scored as 1 = strongly disagree, 2 = disagree, 3 = neutral, 4 = agree, 5 = strongly agree.

## 5 Discussion

This study evaluated the validity and reliability of the ‘Patient Mobilization Attitudes & Beliefs Survey‐ICU’ scale with a sample of 180 healthcare professionals in Turkish healthcare. Language validity was ensured through translation and back‐translation methods, while content validity was established via the Davis method, resulting in a content validity index of 0.87. In confirmatory factor analysis, items 5, 6, 13 and 20 showed non‐significant loadings and were removed, leading to the combination of the knowledge and attitude subscales into a single domain. The resulting two‐factor, 22‐item structure (two subscales: knowledge‐attitude and behaviour) achieved acceptable fit indices and demonstrated good internal consistency reliability. These findings suggest that the adapted Turkish version is a psychometrically sound instrument for assessing healthcare professionals' perceived barriers to mobilisation in intensive care settings.

### 5.1 Construct Validity

The confirmatory factor analysis of the original 26‐item, three‐subscale version of the Patient Mobilization Attitudes & Beliefs Survey‐ICU scale demonstrated poor fit, with items 5, 6, 13 and 20 showing non‐significant regression coefficients (*p* > 0.05). When model fit is not achieved upon testing the existing structure (model), it is recommended to analyse modification suggestions and consider combining the error variances of items within the same subscale. In this study, after testing eight modification suggestions with the existing structure and deleting these items, confirmatory factor analysis of the revised scale (22 items, two subscales) indicated that the fit index values fell within an acceptable/good fit range, supporting its structural validity in a Turkish context.

### 5.2 Reliability

Cronbach's alpha coefficient and the split‐half test method were employed for the reliability assessment of the validated scale. Reliability analysis aims to identify the consistency of responses across scale items. Cronbach's alpha coefficient is commonly utilised to assess the internal consistency of Likert‐type scales. Additionally, inter‐item correlation, item‐total correlation analysis, and the split‐half test offer insights into the internal consistency of the scale. Initial reliability analysis of the 26‐item version showed insufficient internal consistency for the knowledge subscale (*α* = 0.389), while the attitude (*α* = 0.672) and behaviour (*α* = 0.682) subscales, and the total scale (*α* = 0.796), demonstrated satisfactory reliability. The item‐total correlation coefficients of the scale's 5th, 6th, 13th and 20th items, comprising 26 items, fell below +0.25. According to the literature, items with an item‐total correlation below 0.30 do not reflect the conceptual structure. Item‐total correlation values between 0.30 and 0.80 indicate that the items are sufficiently homogeneous and contain the original variance (Sürücü and Maslakçı 2020). Consistent with the literature advocating for removing items with item‐total correlation values below +0.25 (Alpar 2020), the 5th, 6th, 13th and 20th items were removed, and the reliability analysis continued with the 22‐item version featuring two subscales. The Cronbach's alpha coefficients improved to 0.818 (high reliability) for the total scale, 0.721 (quite reliable) for the knowledge and attitude subscales, and 0.708 (quite reliable) for the behaviour subscale. Removing the relevant items resulted in a significant increase in Cronbach's alpha values for both the subscales and the total scale. This finding supports the idea that the removed items weakened the scale's internal consistency. Split‐half reliability coefficients (Spearman‐Brown = 0.799; Guttman = 0.782) further confirmed that the scale was deemed a valid and reliable measurement tool.

Scores obtained from the scale were converted to a 0–100 point system, consistent with the original version. Subscale scores were calculated based on their respective items, with higher total and subscale scores indicating greater perceived barriers. In the original scale (Goodson et al. 2020) and subsequent studies using the PMABS‐ICU, no standardised cut‐off points have been proposed for interpreting scores. Instead, researchers have typically relied on comparisons with findings from other studies, subgroup analyses (Goodson et al. 2020; Parker et al. 2022; Yeung et al. 2023; Aljohani et al. 2024), or repeated measurements over time (Parker et al. 2022).

### 5.3 Comparison With the Original Scale

Compared with the original U.S. version (Goodson et al. 2020), which retained three distinct subscales, the Turkish adaptation required merging knowledge and attitude into a single subscale. This reflects contextual differences in professional roles: occupational therapy is not yet fully integrated into Turkish ICU practice, and physiotherapists are fewer, reducing the salience of certain items. The mean scale score in the Turkish sample (50.23 ± 9.82) was higher than the original scale study (34.6 ± 7.2) (Goodson et al. 2020) and other international studies (32 ± 8) (Parker et al. 2022), suggesting that Turkish healthcare professionals perceive greater barriers to mobilisation.

These differences likely reflect the cultural factors, limited mobilisation practices in intensive care, and ambiguities in professional roles. Previous studies have also shown that unclear roles and responsibilities, inadequate training, insufficient understanding of the benefits of early mobilisation, poor interdisciplinary communication, and a lack of leadership hinder consistent mobilisation (Dirkes and Kozlowski 2019; Kaplan et al. 2023; Dubb et al. 2016). Addressing these barriers requires assessment, structured safety guidelines, standardised mobility protocols, interprofessional education and training and active involvement of leaders (Parker et al. 2022; Hodgson et al. 2021). Quality improvement projects with an interdisciplinary approach demonstrated increased patient mobilisation practices in the ICU and improved patient outcomes (Crooks et al. 2024). Our findings highlight that perceived barriers were higher in Türkiye, which may be linked to cultural differences, less established mobilisation practices, and uncertainties regarding professional responsibilities. The unequal representation of healthcare professionals prevented subgroup analyses across disciplines and the evaluation of discipline‐specific perspectives.

Two items (5th and 6th) ‘I understand which patients are appropriate to refer to Physical Therapy’, ‘I understand which patients are appropriate to refer to Occupational Therapy’ from the knowledge subscale, and one item (13th) ‘mobilization of my patients will be more work for physical and/or occupational’ from attitude subscale, all related to referrals to physiotherapy and occupational therapy, were removed during the adaptation process. The knowledge subscale exhibited insufficient internal consistency (*α* = 0.389), likely reflecting the limited number of physiotherapists (*n* = 5) and majority of nurses (*n* = 163) in the study settings, the limited availability of occupational therapists in the clinical setting, and knowledge and attitude differences in team consultation processes within the study settings compared with the original scale, and as stated in previous studies (Dirkes and Kozlowski 2019; Kaplan et al. 2023; Dubb et al. 2016). The literature supports interdisciplinary collaboration to optimise the feasibility and effectiveness of early mobilisation programmes in intensive care units (Hodgson et al. 2021), however, unlike the original scale, the absence of a multidisciplinary team in the clinics where the study was conducted may lead to gaps in knowledge and practice levels; therefore, we believe that the items should be removed from the scale.

Another item (20th) ‘Family members of my patients are frequently interested to help mobilize them’ was removed from the behaviours subscale during the adaptation process, due to the limited engagement of families in patient care within intensive care units. Although family involvement and support are emerging as integral components of early mobilisation (Singam 2024; Najjar et al. 2021), contextual barriers are constraining the practice in Türkiye. A study in Türkiye reported that 9.6% of intensive care unit nurses involved family members in patient care for educational purposes, with the majority citing institutional protocols (31.7%), concerns about infection, privacy issues and potential disruption of nursing services as obstacles (Özmen et al. 2021). Similar findings have been reported by Aljohani et al. (2024), where family involvement was also identified as a barrier to mobilisation. These system‐level and cultural factors likely explain the poor performance of the family involvement item in the Turkish adaptation. Removing these items, therefore, strengthened the internal consistency and contextual fit of the Turkish version.

Nurses can easily integrate early mobilisation into their routine patient care in the intensive care unit. When nurses are able to provide early mobilisation, significant improvements in patient recovery occur (Liew et al. 2021), leading to faster, healthier recovery and improved overall quality of hospital care (Kaplan et al. 2023). However, although many intensive care nurses are aware of the benefits of early mobilisation, it is not a common practice in ICUs (Liew et al. 2021). Nurses often fear injury to patients and worsening of their condition, largely due to a lack of knowledge among nurses about how to safely perform early mobilisation (Kaplan et al. 2023). Lack of training is a frequently highlighted systemic obstacle (Venema et al. 2025); for instance, Leong et al. (2017) and Zhang et al. (2021) reported that 72% and 52.3% of nurses, respectively, had not received early mobilisation training. In our study, the finding that more than half of the participants had not received formal training on mobilisation aligns with these global trends. This lack of structured education likely played a primary role in the high barrier scores observed, particularly in the knowledge and attitude domains. Intensive care nurses have a low level of knowledge (6.89 ± 2.91) regarding early mobilisation, and most exhibit negative or neutral attitudes towards mobilisation. Nurses who receive training exhibit significantly higher levels of knowledge and more positive attitudes compared to those who do not (Zhang et al. 2021). Furthermore, expertise plays a protective role, as team members with over 10 years of experience often report fewer implementation challenges (Goodson et al. 2020). In our context, the scarcity of regular mobilisation training in nursing programmes and hospitals suggests that perceived barriers may be influenced by individual self‐efficacy perceptions. This may have contributed to higher perceived levels of barriers, particularly in the knowledge and attitude dimensions. Furthermore, the requirement of at least 3 months of ICU experience may have served as a mitigating factor in this context. While formal training was limited, this period of clinical exposure likely provided nurses with a baseline level of ‘situational awareness’ and familiarity with ICU‐specific workflows. This basic clinical immersion might have partially offset the lack of structured education, preventing even higher barrier perceptions by allowing nurses to navigate the environment with at least a foundational understanding of patient safety. The interaction between clinical experience and the absence of formal training suggests that while experience provides a safety net, it cannot fully replace the need for standardised mobilisation education.

The removal of items 5, 6, 13 and 20 was driven by both statistical analyses and contextual and structural considerations specific to Turkish intensive care units. Items 5, 6 and 13, related to multidisciplinary collaboration, exhibited low factor loadings. This finding appears to reflect the current organisational features of Turkish ICUs. In routine practice, the number of physiotherapists assigned to intensive care units is limited. Additionally, no occupational therapists were present in the intensive care units where the study data were collected; the sample primarily consisted of nurses. These structural characteristics likely reduced the relevance and variability of items assessing interdisciplinary collaboration within the study context. In addition, most participants (55.6%) lacked training in mobilisation, a finding supported by the literature as a barrier to implementation. Although evidence‐based protocols and guidelines have been developed to ensure the safety and appropriateness of early mobilisation in intensive care units (Hodgson et al. 2021), no standardised, widely implemented protocol exists across institutions. Early mobilisation is usually initiated according to unit‐specific protocols. Half of the participants reported that their hospitals lacked a formal mobilisation protocol. This lack of institutional standardisation may constitute an additional barrier, particularly in assessing the patient's readiness to initiate early mobilisation (Leong et al. 2017). These findings support that mobilisation is not systematically implemented at the institutional level. Item 20, regarding family involvement, performed poorly in psychometric performance. In Türkiye, family participation in care is often restricted due to institutional protocols, infection control measures, privacy considerations and high workload. Furthermore, families are sometimes perceived as potentially disruptive to workflow. As a result, the construct measured by the item may not adequately reflect routine practice within the study setting. Given that the items did not sufficiently reflect the actual functioning of early mobilisation practices in intensive care units in Türkiye and therefore contributed little to the overall factor scale structure, their removal enhanced the construct validity and contextual relevance of the scale.

### 5.4 Clinical Implications

Mobilisation in intensive care settings is a collaborative effort involving various healthcare professionals, including physical therapists, nurses, physiotherapists and occupational therapists. This interdisciplinary approach, characterised by active joint range of motion (ROM) exercises leading to full ambulation, necessitates continuous engagement from all disciplines (Kaplan et al. 2023). Despite the growing body of evidence highlighting the advantages and feasibility of early mobilisation in intensive care, its widespread adoption remains limited (Goodson et al. 2020). Therefore, recognising and addressing barriers to early mobilisation is imperative for its effective implementation (Singam 2024). Apart from physiological concerns that may impede patient mobilisation, studies have underscored the significance of perceived barriers among healthcare professionals, including factors related to knowledge, attitudes and behaviours (Akhtar and Deshmukh 2021; Goodson et al. 2020; Lewis et al. 2023).

Identification of barriers to patient mobilisation provides critical insight for the development of future effective quality improvement projects, permitting attention to items with barriers of the highest severity (Crooks et al. 2024). The starting point of any project is a unit‐specific evaluation of barriers, emphasising the importance of using a tool, such as the PMABS‐ICU, to understand barriers (Goodson et al. 2020). Assessing barriers to mobility is an essential component of successful implementation of mobility programmes, given that barriers might differ between ICUs (Parker et al. 2022). It is crucial to develop ICU early mobility protocols and training programmes that support clinical needs for safe ICU mobilisation and promote interdisciplinary practice. They should also address the potential barriers and facilitators for early mobilisation (Aljohani et al. 2024). According to the results of a study in which a nurse‐led early mobilisation project was implemented in two intensive care units, the length of stay of patients after the intervention (5.33 days) was reduced compared to patients before the intervention (6.22 days) (Hill 2022). Furthermore, developing mobility‐related measures integrated into clinical care facilitates setting patient goals, monitoring progress, effectively allocating resources and evaluating structured quality improvement programmes (Hodgson et al. 2021).

While numerous studies have explored identifying such barriers and healthcare professionals' perspectives on patient mobilisation (Barber et al. 2015; Jolley et al. 2014; Fontela et al. 2018; Kim et al. 2019; Lewis et al. 2023; Najjar et al. 2021; Yeung et al. 2023; Zhang et al. 2021), the Patient Mobilization Attitudes & Beliefs Survey‐ICU has only been validated in its original English form and, more recently, in Turkish. Some studies (e.g., Parker et al. 2022; Lewis et al. 2023; Aljohani et al. 2024) have used the questionnaire, and Yeung et al. (2023), have used the questionnaire in Singapore ICU settings with minor modifications to the terminologies and included additional demographic questions; no formal linguistic adaptations or psychometric validations have been reported. This highlights a critical gap, as cross‐cultural validation is essential for accurately measuring perceived barriers. The present study addresses this gap by providing the validated adaptation of the scale outside of English, offering a contextually appropriate tool for examining barriers to mobilisation among healthcare professionals in Türkiye. The Turkish version contributes to the international literature by establishing a foundation for future cross‐cultural comparisons, enabling researchers and clinicians to better understand how contextual, cultural, and organisational factors shape healthcare professionals' perceptions of mobilisation worldwide.

### 5.5 Strength and Limitations of the Work

This study has several limitations. First, data were obtained through self‐report, which may have introduced reporting bias. Second, since permission was obtained from only three institutions due to the permission policies of the institutions, the sample size was limited, which may limit the generalizability of the findings. Third, the sample included a disproportionately high number of nurses (*n*: 163) compared with physicians (*n*: 39) and physiotherapists (*n*: 5), which may reduce the applicability of the results to other professional groups. Fourth, since participants could not be retested under the same conditions and time interval, test–retest reliability could not be assessed. Finally, the fact that more than half of the participants had not received formal training in ICU patient mobilisation may represent a significant factor affecting the perceived level of barriers. Since this educational variable was not controlled for in our analysis, it limits the interpretation and generalizability of the findings, as higher barrier scores may partially reflect a lack of baseline technical competency. Despite these limitations, the study provides important insights into the adaptation and validation of a tool to evaluate barriers perceived by healthcare professionals regarding mobilisation in intensive care.

### 5.6 Recommendations for Further Research

Future studies should aim to include multi‐centre and multi‐professional samples, use longitudinal designs, and incorporate test–retest evaluations to strengthen further the evidence for the scale's reliability and validity. Additionally, future research may compare barriers among professionals with varying levels of experience and formal training to better understand how expertise and education influence mobilisation practices.

## 6 Conclusion

The study encompassed a validation and reliability assessment of the ‘Patient Mobilization Attitudes & Beliefs Survey‐ICU’ scale tailored for the Turkish context. Following rigorous validity and reliability analyses and subsequent scale refinements, the revised structure of the ‘Patient Mobilization Attitudes & Beliefs Survey‐ICU’ scale now comprises 22 items, delineated into two distinct subscales: knowledge‐attitude and behaviour. This revised scale has demonstrated robust validity and reliability metrics, rendering it a valid and reliable instrument tailored for assessing attitudes and beliefs pertaining to patient mobilisation within the Turkish society.

## Author Contributions

**Sevgi Uzuntepe:** conceptualisation, formal analysis, investigation, methodology, visualisation, writing – original draft. **Imatullah Akyar:** conceptualisation, methodology, writing – review and editing, supervision, visualisation.

## Funding

The authors have nothing to report.

## Disclosure

Statistics: The statistics were checked prior to submission by Mustafa Sabri Kovancı. His email address is msabri.kovanci@gmail.com.

## Conflicts of Interest

The authors declare no conflicts of interest.

## Data Availability

The data that support the findings of this study are available from the corresponding author upon reasonable request.
